# *Rostania* revised: testing generic delimitations in Collemataceae (Peltigerales, Lecanoromycetes)

**DOI:** 10.3897/mycokeys.47.32227

**Published:** 2019-02-20

**Authors:** Alica Košuthová, Martin Westberg, Mónica A.G. tálora, Mats Wedin

**Affiliations:** 1 Department of Botany, Swedish Museum of Natural History, P.O. Box 50007, SE-104 05 Stockholm, Sweden Swedish Museum of Natural History Stockholm Sweden; 2 Museum of Evolution, Uppsala University, Norbyvägen 16, SE-752 36, Uppsala, Sweden Uppsala University Upssala Sweden; 3 Plant Ecological Genetics, Institute of Integrative Biology, ETH Zurich, Universitätstrasse 16, 8092 Zurich, Switzerland Institute of Integrative Biology Zurich Switzerland

**Keywords:** Classification, cyanolichens, nomenclature, systematics, taxonomy, thallus anatomy

## Abstract

Here, we test the current generic delimitation of *Rostania* (Collemataceae, Peltigerales, Ascomycota) utilizing molecular phylogeny and morphological investigations. Using DNA sequence data from the mitochondrial SSU rDNA and two nuclear protein-coding genes (MCM7 and β-tubulin) and utilizing parsimony, maximum likelihood and Bayesian phylogenetic methods, *Rostania* is shown to be non-monophyletic in the current sense. A new generic delimitation of *Rostania* is thus proposed, in which the genus is monophyletic, and three species (*Rostaniacoccophylla*, *R.paramensis*, *R.quadrifida*) are excluded and transferred to other genera. *Rostaniaoccultata* is further non-monophyletic, and a more detailed investigation of species delimitations in *Rostania* s. str. is needed. The new combinations *Leptogiumparamense* and *Scytiniumquadrifidum* are proposed.

## Introduction

Collemataceae is a large group of predominantly foliose lichenized fungi commonly known as the “jelly lichens” due to their gelatinous habit. This is caused by a polysaccharide matrix around the *Nostoc* cyanobacterial photobionts that swells and becomes extremely gelatinous when wet. Until very recently, the generic classification of the Collemataceae s. str. was very unnatural and based solely on one character, presence (*Leptogium*) or absence (*Collema*) of a cellular cortex (Degelius 1954, 1974; Jørgensen 2007). Already Degelius (1954) questioned the monophyly of *Collema* and *Leptogium*. This was also supported by molecular phylogenies (Wiklund and Wedin 2003; Miadlikowska and Lutzoni 2004; Miadlikowska et al. 2014), and somewhat surprisingly, gelatinous genera with one-septate spores that earlier were classified in Collemataceae, were shown to belong to the Pannariaceae (Wedin et al. 2009; Otálora et al. 2010; Ekman et al. 2014; Weerakoon et al. 2018) or Arctomiaceae (Otálora and Wedin 2013). Not until Otálora et al. (2013a, 2013b) investigated the family in detail was a modern classification of Collemataceae s. str. proposed. *Collema* and *Leptogium* were confirmed as highly non-monophyletic, and Otálora et al. (2013b) instead suggested accepting 10 more or less morphologically distinct monophyletic groups from their tree, as genera. In addition to *Collema* and *Leptogium* in restricted senses, six old generic names were resurrected (*Blennothallia* Trevis., *Enchylium* (Ach.) Gray, *Lathagrium* (Ach.) Gray, *Pseudoleptogium* Müll. Arg., *Rostania* Trevis., and *Scytinium* (Ach.) Gray), and two new genera were described (*Callome* Otálora & Wedin and *Paracollema* Otálora & Wedin).

*Rostania*, the focus of the present study, corresponds to the *Occultatum*-group of *Collema* (Degelius 1954, 1974). It is a comparatively small genus with eight currently accepted, mainly epiphytic species, characterised by very small to medium sized (ca 0.3–5 cm in diam.) subcrustaceous to subfoliose thalli with very small apothecia (ca 0.2–0.8(–1) mm in diam.) and cuboid to oblong muriform spores. All five species included in the *Occultatum* group by Degelius were treated in *Rostania* by Otálora et al. (2013b); *Rostaniacallibotrys* (Tuck.) Otálora, P.M. Jørg. & Wedin, *Rostaniaceranisca* (Nyl.) Otálora, P.M. Jørg. & Wedin, *Rostaniacoccophylla* (Nyl.) Otálora, P.M. Jørg. & Wedin, *Rostaniaoccultata* (Bagl.) Otálora, P.M. Jørg. & Wedin and *Rostaniamultipunctata* (Degel.) Otálora, P.M. Jørg. & Wedin. In addition, *Rostanialaevispora* (Swinscow & Krog) Otálora, P.M. Jørg. & Wedin was included in the genus. Two further species were later added, *Rostaniaparamensis* (P.M. Jørg. & Palice) P.M. Jørg. & Palice (Jørgensen and Palice 2015) and *Rostaniaquadrifida* (D.F. Stone & McCune) McCune (McCune et al. 2014). Degelius (1954) divided *Collemaoccultatum* into two varieties: var. populinum which was characterised by a squamulose, somewhat lobate thallus, and which almost exclusively grew on the bark of *Populus*, and var. *occultatum* with a granulose thallus and which occurred on many deciduous trees, including *Populus*. Perlmutter and Rivas Plata (2018) combined var. populinum in *Rostania*, as R.occultatavar.populina (Th. Fr.) Perlmutter & Rivas Plata.

Otálora et al. (2013a, 2013b) included only three species (*R.ceranisca, R.multipunctata* and *R.occultata*) in their phylogenies, and thus the taxonomical position of most species has not been tested by molecular methods. As there is a substantial variation in shape and size of the lobes, apothecia and ascospores, as well as the hyphal arrangement in the thallus among the *Rostania* species, and as several former Collemataceae taxa have been shown to belong outside the family, the delimitation of the whole genus needs investigation. Here, we will test the generic delimitation of *Rostania* and investigate the relationships of any species falling outside *Rostania* s. str. Finally, we will note and comment on any indication of species non-monophyly, in this genus.

## Material and methods

### Taxon sampling and morphological studies

We sampled 52 specimens of Collemataceae for the molecular study, including six of the eight currently accepted *Rostania* species and representatives of all genera within the family Collemataceae, including type species. Sequences originating from the study of Otálora et al. (2013a) were downloaded from GenBank (https://www.ncbi.nlm.nih.gov/) and all sequences used in this work are summarized in Table 1. Our own collections were deposited in UPS and S, and additional herbarium material from the herbaria PRA, GZU, UPS and S was also included (Table 1). Additional herbarium type material from the herbaria H and O was studied morphologically only (listed on the end of the manuscript). Herbarium acronyms follow Thiers (2018). Three species of *Rostania* not included in earlier studies were successfully added (*R.callibotrys*, *R.quadrifida* and *R.paramensis*). The sampling of *Rostaniaoccultata* included specimens of both varieties. To enable testing of generic monophyly and family placement of taxa potentially to be excluded from *Rostania*, we added secondary outgroups including newly produced sequences of two species from the sister family Placynthiaceae (*Placynthiumnigrum* and *P.rosulans*) and sequences available in GenBank of two from the more distantly related Pannariaceae (*Pannariarubiginosa* and *Staurolemmaomphalarioides*). Finally, *Peltigeraaphthosa* was used as outgroup to root the tree.

**Table 1.** Sequences utilized in this study (newly produced sequences in bold, remaining sequences produced by Otálora et al. (2013a) and some of the outgroup sequences are taken from Wiklund and Wedin (2003), Buschbom and Mueller (2004), Otálora et al. (2010), Prieto et al. (2013)). In case of *Rostania* species, origin of both, state and provinces are given.

Taxon	Geographic origin, voucher	GenBank accession number		
		mtSSU	b-tub	MCM7
*Blenothallia crispa1*	Hungary: Thor 7021a (UPS–L48439)	JX992918	KC119040	JX992976
*Blenothallia crispa 3*	Spain: Westberg (S–F315217)	**MK445278**	**MK451934**	**MK451920**
*Callome multipartita 1*	Norway: Haugan 7015 (O–L117369)	GQ259019	–	–
*Callome multipartita 2*	Austria: Hafellner 74818 (GZU18–2009)	**MK445271**	**MK451935**	–
* Collema leptaleum *	Argentina: Wedin 8822 (S–F335749)	JX992928	KC119038	JX992986
* Collema nigrescens *	Spain: Aragón 80/04 (MA–16262)	EU982563	KC119016	JX992989
* Collema subconveniens *	New Zealand: Wedin 9225 (S–F335747)	JX992937	KC119019	JX992996
* Enchylium bachmanianum *	Sweden: Nordin 1521 (UPS–L133627)	JX992914	**MK451936**	JX992974
*Enchylium polycarpon 3*	Sweden: Odelvik 04700 (S–L316455)	JX992934	**MK451937**	JX992993
*Enchylium tenax 1*	Spain: Etayo 20214 (MA–L13396)	EU982556	KC128823	JX992998
*Enchylium tenax 2*	Spain: Sarrión 1509 (MA–L14789)	EU982579	KC128824	–
* Lathagrium auriforme *	Spain: Otálora 20904 (MA–L16249)	JX992913	KC119008	JX992973
* Lathagrium cf. fuscovirens *	Sweden: Wedin 9701 (S–F332476)	**MK445277**	**MK451938**	**MK451921**
* Lathagrium fuscovirens *	Sweden: Tibell 23588 (UPSL–145162)	JX992923	KC119013	JX992983
* Leptogium azureum *	Chile: Cornejo 26507 (MA–16273)	JX992939	KC119021	JX993002
* Leptogium byssinum *	Norway: Westberg (S–F264803)	KT240180	–	KT240183
* Leptogium denticulatum *	Argentina: Wedin 8690 (S–F332474)	JX992947	KC119025	JX993012
* Leptogium terrenum *	Portugal: van den Boom 41781 (hb. van den Boom)	KT240181	–	KT240184
*Paracollema italicum 1*	Croatia: Nordin 2708 (UPS–L076283)	JX992925	KC119015	JX992984
*Paracollema italicum 3*	Croatia: Nordin 2763 (UPS–L076284)	JX992926	–	JX992985
*Pseudoleptogium diffractum 1*	Sweden: Nygren 007 (UPS–L129612)	GQ259029	–	–
*Pseudoleptogium diffractum 3*	Sweden: Nordin 2529 (UPS–L153952)	JX992949	–	JX993015
*Rostaniacallibotrys 1*	Kenya: Moberg 4431a (UPS–L22044)	**MK445270**	**MK451939**	–
*Rostaniacallibotrys 2*	Costa Rica: Sipman 20495 (GZU–113_8P)	**MK445269**	**MK451940**	–
*Rostaniaceranisca 1*	Norway, Troms: Nordin 5721 (UPS–L130978)	**MK445280**	**MK451941**	–
*Rostaniaceranisca 2*	Sweden, Pite Lappmark: Westberg PL433 (UPS-L931677)	**MK445267**	**MK451942**	**MK451922**
*Rostaniaceranisca 3*	Austria, Salzburg: MW_HOCH020 (S–F262465)	**MK445268**	**MK451943**	**MK451923**
*Rostaniamultipunctata 1*	Greece, Crete: Nordin 3160 (UPS–L027750)	JX992930	**MK451944**	JX992988
*Rostaniamultipunctata 2*	Greece, Korfu: Poelt 8852 (GZU–2–93)	**MK445273**	**MK451945**	–
*Rostaniaoccultatav.occultata 1*	Sweden, Pite Lappmark: Westberg PL467 (UPS-L931673)	**MK445266**	**MK451946**	**MK451924**
*Rostaniaoccultatav.occultata 2*	Sweden, Dalarna: Westberg (S–F304739)	**MK445259**	–	**MK451925**
*Rostaniaoccultatav.occultata 3*	Sweden, Uppland: Westberg (UPS–L834451)	**MK445257**	–	**MK451926**
*Rostaniaoccultatav.populina 1*	Sweden, Södermanland: Nordin 5407 (UPS–L120396)	JX992931	–	JX992991
*Rostaniaoccultatav.populina 2*	Greece, Crete: Llop 56060303 (S–F233720)	JX992932	**MK451947**	JX992990
*Rostaniaoccultatav.populina 3*	Sweden, Gästrikland: Odelvik 01269 (S–L42490)	**MK445260**	**MK451948**	**MK451927**
*Rostaniaoccultatav.populina 4*	Sweden, Jämtland: Kosuthova 174 (S–F332481)	**MK445265**	**MK451949**	**MK451928**
* Rostania paramensis *	Ecuador, Carchi: Palice 2796 (PRA–00013999) (HOLOTYPE)	**MK445279**	–	–
*Rostaniaquadrifida 1*	USA, Oregon: McCune 2744 (UPS–L513233)	**MK445272**	**MK451950**	–
*Rostaniaquadrifida 2*	USA, Oregon: McCune 28536 (UPS–L513222) (ISOTYPE)	**MK445274**	**MK451951**	–
* Scytinium biatorinum *	Sweden: Jonsson 5500 (UPS–L186460)	JX992940	KC119022	JX993003
* Scytinium imbricatum *	Sweden: Hermansson 18777 (UPS–L706500)	**MK445264**	**MK451952**	**MK451929**
* Scytinium intermedium *	Sweden: Nordin 7385 (UPS–L587203)	**MK445263**	**MK451953**	**MK451930**
* Scytinium magnussonii *	Spain: Otálora 20104 (MA)	EU982565	KC119004	JX993022
* Scytinium palmatum *	Sweden: Nordin 5369 (UPS–L113313)	JX992959	KC119027	JX993025
* Scytinium parvum *	Sweden: Thor 4300 (UPS–L174011)	JX992933	KC119018	JX992992
* Scytinium plicatile *	Sweden: Nordin 5566 (UPS–L124847)	GQ259033	KC119030	JX993030
* Scytinium pulvinatum *	Russia: Pystina 17352 (UPS–L738570)	**MK445262**	**MK451954**	**MK451931**
*Scytinium* sp_Palice2273	Ecuador: Palice 2273 (PRA–00013997)	**MK445275**	**MK451955**	–
*Scytinium* sp_Palice2274a	Ecuador: Palice 2274a (PRA–00013998)	**MK445276**	–	–
* Scytinium subtile *	Sweden: Ågren 686 (UPS–L163890)	JX992869	KC119034	–
* Scytinium tenuissimum *	Spain: Aragón 1682/97 (MA)	JX992971	KC119036	–
* Scytinium turgidum *	Spain: Aragón 1671/98 (MA–12868)	EU982592	KC119037	JX993040
**Outgroups**:				
* Placynthium rosulans *	Sweden: Westberg URL222 (UPS–L854413)	**MK445258**	**MK451956**	**MK451932**
* Placynthium nigrum *	Sweden: Kosuthova 35 (S–F332479)	**MK445261**	–	**MK451933**
* Pannaria rubiginosa *	Portugal: Purvis et Smith 27/4/95 (BM)	AY340513	–	JX993042
* Staurolemma omphalarioides *	Spain: Aragón 83/04 (MA), mtSSU only Spain: Hafellner & Hafellner 41399 (UPS), MCM7 only	EU982560	–	JX993043
* Peltigera apthosa *	Sweden: Wedin 6164 (UPS)	AY340515	AY536792	JX000176

We studied morphological and anatomical characters under the light microscope and dissecting microscope. We used hand-cut longitudinal sections of apothecia to observe internal and microscopic characteristics, in water. Microscopic examinations of the thalli were conducted on transversal cross-sections of lobes in water, or lactic blue.

### Data generation

Two apothecia or (in the case of sterile samples) a thallus fragment, were selected for extraction. We extracted total DNA using the Plant DNA Mini Kit (Qiagen, Hilden, Germany) following the manufacturers’ instructions. We amplified ca 0.6 kb of the small subunit of the mitochondrial rDNA (mtSSU), ca 0.6 kb of the two protein-coding genes DNA replication licensing factor mini-chromosome maintenance complex component 7 (MCM7) and the β-tubulin gene (b-tub) using the same primer combinations and PCR settings as in previous studies (Otálora et al. 2013a; Košuthová et al. 2016). We assembled and edited DNA sequences using Geneious version R8 (http://www.geneious.com; Kearse et al. 2012).

### Sequence alignment and analysis

To identify and avoid contaminants among the new sequences, we used Megablast high similarity matches in Geneious version R8 (http://www.geneious.com; Kearse et al. 2012). Alignments were constructed using AliView 1.09 (Larsson 2014) with the “ClustalW/Multiple alignment” option and subsequent manual adjustments. All ambiguously aligned regions (sensu Lutzoni et al. 2000) were excluded from analysis.

The mitochondrial and the two protein-coding datasets were analysed separately before concatenation using parsimony jackknifing (JK) in WinClada (Nixon 1999–2002) with 100–200 replicates and otherwise default settings. As no significant (JK support above 70%) incongruence was detected, the alignments were concatenated. Final alignments have been deposited in TREEBASE (http://www.treebase.org) with accession number (http://purl.org/phylo/treebase/phylows/study/TB2:S23889). After concatenation, we inferred phylogenetic relationships using parsimony, maximum likelihood and Bayesian phylogenetic methods with indels treated as missing data. Partitions scheme and optimal model of nucleotide substitution for Bayesian analysis were selected using PartitionFinder2 (Guindon et al. 2010; Lanfear et al. 2012, 2016). PartitionFinder was set as follow: linked branch lengths, data blocks according to each codon position of each genetic region (mtSSU, MCM7, b-tub), the greedy search scheme, the Bayesian information criterion as selection metric and only models that are implemented in MrBayes. The selected substitution model schemes are provided in Table 2.

We performed parsimony JK in WinClada (Nixon 1999–2002) with 2000 replicates and otherwise default settings. For maximum likelihood and ML bootstrapping we used RAxML 8 (Stamatakis 2014) implementing a general time reversible (GTR) model of nucleotide substitution with gamma distributed rate heterogeneity GTR+G (GTRGAMMA)following recommendations in the user manual. We used 4 partitions determined by PartitionFinders (Table 2). 1000 bootstrap (BS) replicates were completed using the parametric (BS) algorithm of RAxML-HPC2 on the Cipres Web Portal (Miller et al. 2010). Bayesian phylogenetic analysis was inferred using MrBayes 3.2.5 (Huelsenbeck and Ronquist 2001; Ronquist and Huelsenbeck 2003; Ronquist et al. 2011) with the evolutionary models following the partitioning scheme from PartitionFinder (Table 2). We estimated posterior probabilities (PP) by running one cold and two heated chains for 2 130 000 generations in parallel mode, saving trees every 100^th^ generation. To test whether the Markov chain converged, we monitored the average standard deviation of split frequencies (ASDSF), which should fall below 0.01 when comparing two independent runs. We discarded the 25% of generations before the point where the ASDSF fell below 0.01 as burn-in. All remaining trees were summarized as a Bayesian 50% majority rule (MR) consensus tree with PP calculated for each clade.

**Table 2.** Evolutionary models and partitions according to the Best scheme calculated in PartitionFinder. In RAxML only the GTR+G (GTRGAMMA) model was used for all partitions.

Subset name	Analyses type	Nr of sites	Codon position	Best model	Partition
mtSSU	MrBayes	735	–	HKY+I+G	1
MCM7	MrBayes	194	1	SYM+I+G	2
MCM7	MrBayes	194	2	SYM+I+G	2
MCM7	MrBayes	194	3	HKY+I+G	3
b-tub	MrBayes	210	1	SYM+I+G	2
b-tub	MrBayes	210	2	JC	4
b-tub	MrBayes	210	3	HKY+I+G	3
mtSSU	RAxML	735	–	–	1
MCM7	RAxML	194	1	–	2
MCM7	RAxML	194	2	–	3
MCM7	RAxML	194	3	–	4
b-tub	RAxML	210	1	–	2
b-tub	RAxML	210	2	–	3
b-tub	RAxML	210	3	–	4

## Results and discussion

**Figure 1.**
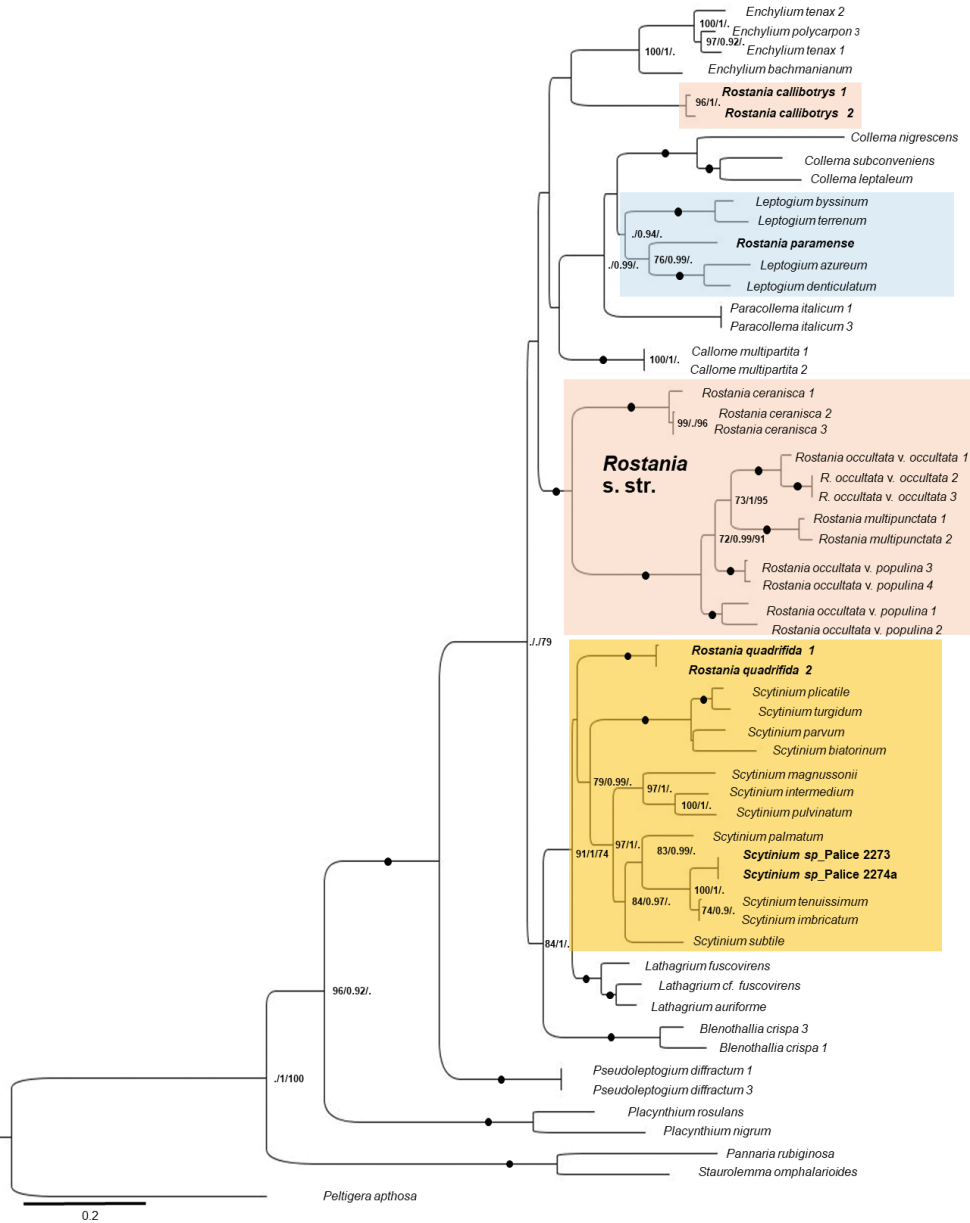
The most likely tree from the combined RAxML analysis based on 1947 aligned characters of mtSSU rDNA, MCM7 and b-tub from 57 specimens. Support values (Likelihood BS/Bayesian PP/parsimony JK) given when BS ≥ 70%, PP ≥ 0.90 and parsimony JK ≥ 70%. Branches receiving BS ≥ 75 %, PP ≥ 0.95 and JK ≥ 75% are indicated with a black dot. The different colour indicate different genera: blue = *Leptogium*, pink = *Rostania*, orange = *Scytinium*.

We produced 61 new sequences (Table 1) for the phylogenetic analyses (24 mtSSU, 15 MCM7, 22 b-tub) including 57 taxa and 1947 nucleotide positions (735 for mtSSU and 582 for MCM7 and 630 for b-tub) for the final matrix. The alignment contained 618 parsimony-informative characters (177 for mtSSU, 237 for MCM7 and 204 for b-tub). The most likely tree from the RAxML analysis is presented in Figure 1 with likelihood BS, Bayesian PP and parsimony JK support superimposed.

**Figure 2.**
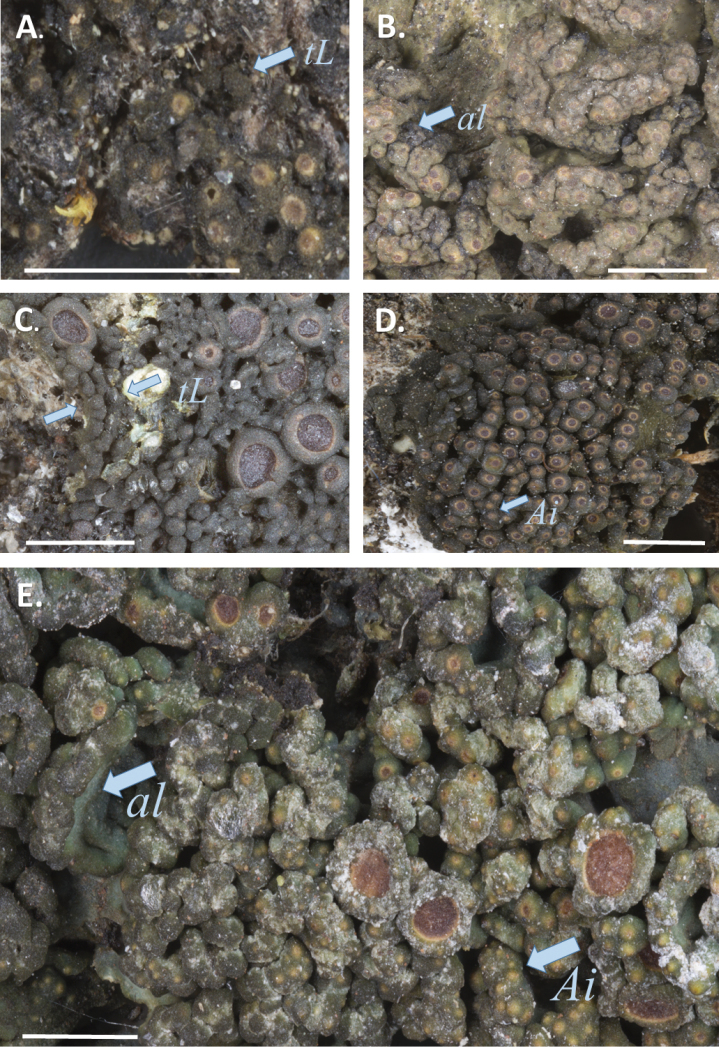
Thallus habitus: **A**Rostaniaoccultatavar.populina (Odelvik 1269, S), thallus lobes (arrow) **B***Rostaniamultipunctata* (Poelt 8852, GZU), accessory lobules (arrow) **C***Rostaniaceranisca* (MW_HOCH020, S), accessory finger-like lobules (arrow) **D***Rostanialaevispora* (isotype of *Collemalaevisporum* Swinscow & Krog, Tanzania, 1986, Swinscow & Krog T 3/6, O-00298), apothecium in initial stage (arrow) **E***Rostaniacallibotrys* (Moberg 4431a, UPS), apothecium in initial stage (arrow). ***tL*** = thallus lobes, ***al*** = accessory lobules, ***Ai*** = apothecium in initial stage covering the top of the accessory lobules. Scale bar: 1 cm.

**Figure 3.**
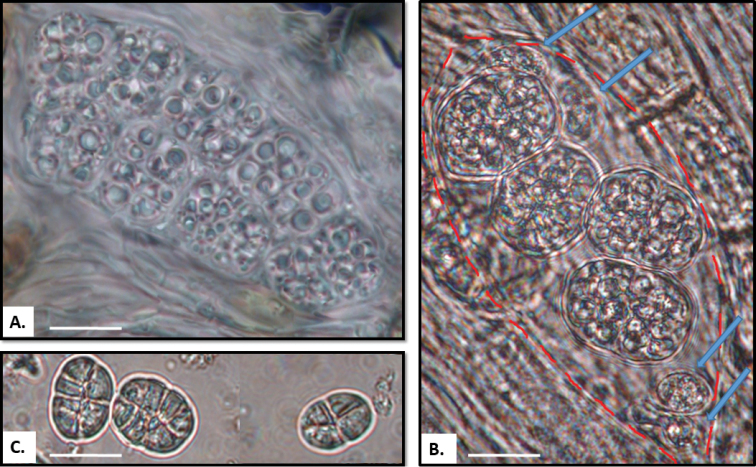
Ascospores: **A**Rostaniaoccultatavar.populina (Llop 56060303, S), cubic spores **B***Rostaniaceranisca* (Westberg L271_PL433, UPS), oblong spores; ascus (red line) with only four mature spores visible but remnants of four aborted spores can be seen (arrows) **C***Rostaniacallibotrys* (Sipman 2049, GZU), oblong spores. Scale bar: 10 µm.

The analyses resulted in a topology (Fig. 1) very similar to the results of Otálora et al. (2013a, 2013b). Some of the backbone topology, however, has unfortunately no or low support. In Otálora et al. (2013b)*Callome* was the sister to *Rostania*, but in our study this relationship is not formed. All *Rostania* species are nested within Collemataceae, but *Rostania* in the sense of Otálora et al. (2013b) is non-monophyletic. Three species form a core group, which we here treat as *Rostania* s. str. *Rostania* s. str. is well supported and includes *Rostaniaoccultata* (Fig. 2A), *R.ceranisca*, and *R.multipunctata*. We can conclude that *R.occultata* as currently circumscribed is non-monophyletic. *Rostaniamultipunctata* (Fig. 2B) shares the cuboid shape and size of the spores with *R.occultata* s. lat. (Fig. 3A), but the thallus differs in size (the lobes are generally larger, up to ca 2.5 cm long in *R.multipunctata*, while in *R.occultata* s. lat. they are up to ca 3 mm long). It has also accessory lobules developing from the wrinkles (Fig. 2B), which do not occur in *R.occultata* s. lat. The delimitation of the two varieties of *R.occultata* is unclear, as is the separation from *R.multipunctata*. Our study is not designed to study species-delimitations and we will extend our investigation of this species complex in a larger study currently in preparation.

*Rostaniaceranisca*, the only terricolous *Rostania*, is sister to the group consisting of *R.multipunctata* and *R.occultata* s. lat. In addition to its terricolous ecology, it is easily recognized by the erect accessory finger-like lobules (Fig. 2C), which grow from the edge of the main lobes. The spores in *R.ceranisca* differ in shape from the cuboid spores in *R.multipunctata* and *R.occultata* s. lat. (Fig. 3A) in being oblong (Fig. 3B). Although Degelius (1954) noted only four spores in the ascus, we have usually observed eight spores, even if four of them may be aborted or are at least not clearly visible when mature (Fig. 3B).

*Rostaniacallibotrys* does not group with *Rostania* s. str. (Fig. 1), but forms an unsupported group with *Enchylium*. *Rostaniacallibotrys* has a comparatively distinct thalline apothecium margin, similar to some species of *Enchylium*. However, this is a widespread feature in the family including some species of *Rostania* s. str. The thallus with characteristic accessory lobules in *R.multipunctata* (Fig. 2B) and *R.laevispora* (Fig. 2D) is very similar to *R.callibotrys* (Fig. 2E). *Rostaniacallibotrys* also has spores that are very similar to the typical cuboid to oblong *Rostania*-spores in *R.multipunctata* and *R.occultata* s. lat. (Fig. 3A, B), but the spores in *R.callibotrys* have fewer cells (Fig. 3C) than in these species. *Rostanialaevispora* (Fig. 2D), a rarely collected species that we did not manage to get sequences from, is very similar and likely very closely related to *R.callibotrys* (Fig. 2E). As there is no support for excluding these species, and no distinct morphological evidence suggests any other relationship, we tentatively leave both *R.callibotrys* and *R.laevispora* in *Rostania*.

**Figure 4.**
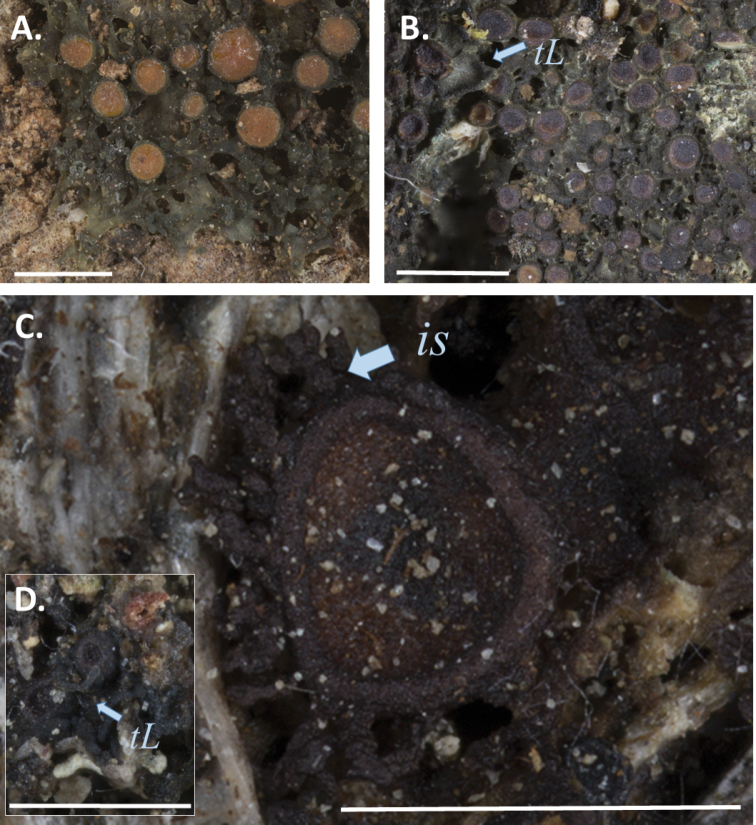
Thallus habitus: **A***Rostaniacoccophylla* (isotype of *Collemacoccophyllum* Nyl., India, 1858, Perrotet s.n., H-NYL 42355, H–9201376) **B***Rostaniaparamensis* (Palice 2796, PRA-00013999; holotype of *Collemaparamense* PM Jørg. & Palice) **C***Scytinium* sp. Palice 2274a **D***Rostaniaparamensis* Palice 2274b. ***tL*** = thalline lobes, ***is*** = isidia. Scale bar: 1 cm.

We did not manage to get molecular data from *R.coccophylla* (Fig. 4A), a tropical and rarely collected species where the available material was too old. Although *R.coccophylla* is similar to *R.callibotrys* and *R.multipunctata*, the apothecia in *R.coccophylla* are very different in that they are convex and stipitate when mature (compared to concave and initially immersed and later sessile, in *Rostania*) and considerably larger compared to other *Rostania* species. The apothecia of *R.coccophylla* are similar to several species in *Collema* sensu Otálora (2013b), where this species originally was placed. Although we preferably would want molecular data to test the correct placement of this species, we suggest that it is re-instated in *Collema*, where the name *Collemacoccophyllum* Nyl. is available.

**Figure 5.**
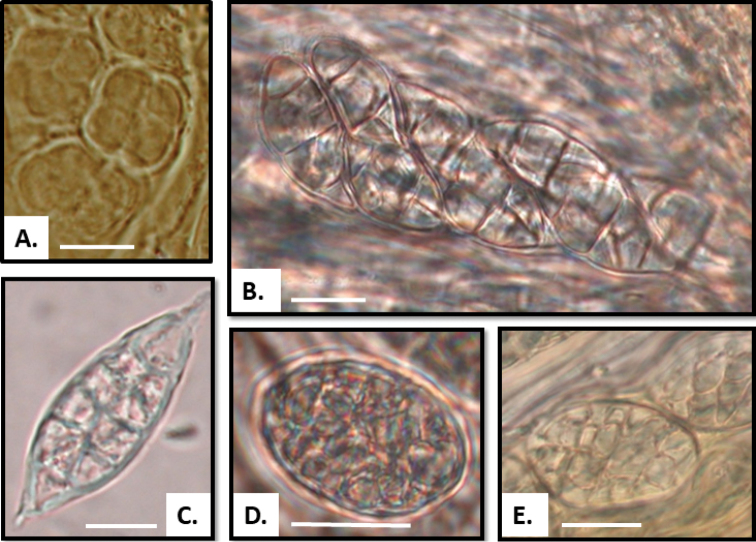
Ascospores: **A***Rostaniaquadrifida* (McCune 2744, UPS), cubic spores with 2–5 cells **B***Rostaniaparamensis* (Palice 2796, holotype of *Collemaparamense*), ellipsoid spores with acute ends **C***Leptogiumazureum* (Tehler 3140, S), ellipsoid spores with acute ends **D***Scytinium* sp. (Palice 2273), oblong spores, **E***Scytinium* sp. (Palice 2274), oblong spores. Scale bar = 10 µm

**Figure 6.**
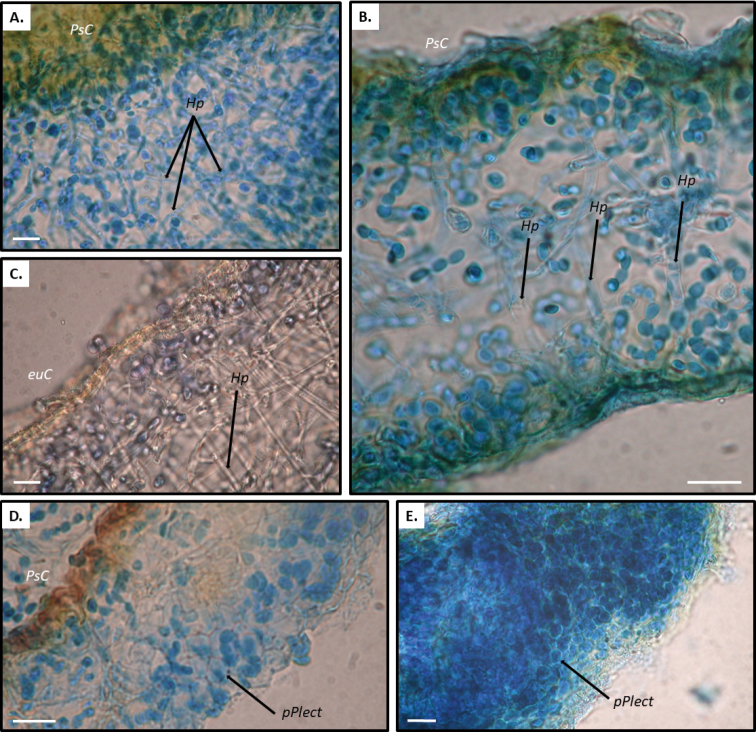
Thalli, transversal cross-sections: **A** Thallus with pseudocortex and densely interwoven hyphae (*Rostaniaquadrifida*, McCune 2744, UPS) **B** Thallus with pseudocortex and straight and unbranched hyphae which are perpendicular to the surface (*Rostaniaparamensis*, Palice 2796, holotype of *Collemaparamense*) **C** Thallus with eucortex and straight and unbranched hyphae which are perpendicular to the surface (*Leptogiumazureum*, Tehler 3140, S) **D** Thallus with eucortex and paraplectenchymateous throughout (*Scytinium* sp. Palice 2273) **E** Thallus paraplectenchymateous throughout (Rostaniaoccultatavar.populina, Llop 56060303, S) **A–E** in lactic blue **C** in water. euC = eucortex, PsC = pseudocortex, Hp = hyphae, pPlect = paraplechtenchyma. Scale bar = 10 µm

*Rostaniaquadrifida* and *R.paramensis* are not closely related to *Rostania* s. str. *Rostaniaquadrifida* was described by Stone and McCune (2010) as *Collemaquadrifidum*, and was later included in *Rostania* based on spore shape and thallus morphology (McCune et al. 2014). It differs from *Rostania* s. str. by having spores with fewer septa (Fig. 5A). Here it forms the sister group to *Scytinium* (Fig. 1), within a well-supported group consisting of *Blennothallia*, *Lathagrium* and *Scytinium*. *Rostaniaquadrifida* has a thallus composed by densely interwoven hyphae, and with a pseudocortex (Fig. 6A), features that do not occur in *Rostania* s. str., but in some species of *Scytinium* (similar to e.g. *Scytiniumintermedium* and *S.magnussonii*; Jørgensen 1994). These similarities support including it in *Scytinium*, which we do below.

The generic position of *R.paramensis* has been complicated to assess. Jørgensen and Palice (2012) described it as *Collemaparamense*, based on the holotype (Palice 2796) and another sample from a second locality in Ecuador (Palice 2273). As the thallus has a pseudocortex, Otálora et al. (2013b) transferred it to *Scytinium*. Jørgensen and Palice (2015) later studied another sample from the second locality (Palice 2274). They concluded that the spores in the holotype must have been unusually developed, and transferred it to *Rostania* based on the oblong spores (similar to *R.ceranisca*) found in Palice 2274. Our re-examination of these three specimens, including the holotype, shows that Palice 2273 and Palice 2274 contain two distinct Collemataceae species (Fig. 4C, D). One of these (Fig. 4D), present in small amounts only in both samples, is identical with holotype of *Collemaparamense* and is characterised by a matt dark olive thallus with a pseudocortex (Fig. 6B), and hyaline, muriform, ellipsoid spores with acute ends (Fig. 5B). This is very different from the spores in *Rostania*, but typical for species in *Leptogium* s. str. (Fig. 5C). We sequenced the holotype, and we can conclude that among the *Leptogium* species we have sampled, it forms a group with *Leptogiumazureum* (the conserved type of *Leptogium*; Jørgensen et al. 2013) and *L.denticulatum* (Fig. 1). It has a thallus which is appressed to the substrate and composed by relatively small lobes (Fig. 4B) which is rare in other Leptogiums. str., and insectionit has straight and unbranched hyphae which are perpendicular to the surface (Degelius 1954; Fig. 6B). This character is present in several groups in Collemataceae. It was observed by Degelius (1954) in some *Collema* species, and has also been noted in the newly described *Leptogiumantarcticum* by Kitaura et al. (2018) who used the term “columnar hyphae” for the same hyphal arrangement. We have observed this hyphal arrangement in *Leptogiumazureum* (Fig. 6C) and *L.denticulatum* too, but it is apparently not present in *Rostania*. The second species present in Palice 2273 and Palice 2274, apparently confused Jørgensen and Palice (2015) as their observation of oblong spores (Fig. 5D, E) refer to this species, which has a shiny brown thallus (Fig. 4C) and not a matt dark olive thallus as in “*Rostania*” *paramense* (Fig. 4B). The second species differs from *Rostania* by having a proper eucortex (Fig. 6D), and by producing isidia along the apothecium margin (Fig. 4C). The thallus is paraplectenchymateous throughout (Fig. 6D). This hyphal arrangement is present in several groups in Collemataceae, including *Rostaniaoccultata* s. lat. (Fig. 6E). Already Degelius (1954) noted this hyphal arrangement in his *Occultatum*-group and Otálora et al. (2013b) observed the same in *Blennothallia*, *Pseudoleptogium* and in *Scytinium*. We sequenced also this species and we can confirm that both samples belong in *Scytinium*, but the species remains to be identified.

## Conclusions

Here we have tested the current generic concept of *Rostania* and conclude that at least three of the species should be excluded and that the position of *R.callibotrys* and *R.laevispora* in *Rostania* is uncertain. *Rostania* is characterized by crustose to subfoliose thallus with initially immersed apothecia (Fig. 2D, E), which only later become sessile. The disc is concave when young and plane when older, but never convex. The spores are muriform with at least 5 cells, cuboid to oblong, but never fusiform to ellipsoid (Fig. 3A–C). Most species are comparatively small, and all lack cortex, rhizines and isidia.

*Rostania* includes six taxa: *R.callibotrys*, *R.ceranisca*, *R.laevispora*, *R.multipunctata*, R.occultatavar.occultata, and R.occultatavar.populina. *Rostaniaoccultata* s. lat. is non-monophyletic and this species complex will be investigated in the near future.

### New combinations

#### Leptogium paramense

Taxon classificationFungiPeltigeralesCollemataceae

(P.M.Jørg. & Palice) A.Košuth. & Wedin comb. nov.

829590

##### Basionym.

*Collemaparamense* P.M. Jørg. & Palice, Biblioth. Lichenol. 108: 136 (2012)

**Type.** ECUADOR. Carchi: volcan Chiles, wet paramo, Palice 2796 (PRA-00013999!–holotype, BG, QCA–isotypes).

#### Scytinium quadrifidum

Taxon classificationFungiPeltigeralesCollemataceae

(D.F.Stone & McCune) A.Košuth. & Wedin comb. nov.

MB829591

##### Basionym.

*Collemaquadrifidum* D.F. Stone & McCune, *N. Amer. Fung.* 5(2): 2 (2010)

##### Type.

U. S. A. OREGON, Douglas County: Bushnell-Irwin Rocks ACEC, McCune 28536 (OSC–holotype, US, UPS–L513222!–isotypes).

## Supplementary Material

XML Treatment for Leptogium paramense

XML Treatment for Scytinium quadrifidum
